# Identification of Colon Immune Cell Marker Genes Using Machine Learning Methods

**DOI:** 10.3390/life13091876

**Published:** 2023-09-07

**Authors:** Yong Yang, Yuhang Zhang, Jingxin Ren, Kaiyan Feng, Zhandong Li, Tao Huang, Yudong Cai

**Affiliations:** 1 Qianwei Hospital of Jilin Province, Changchun 130012, China; yqngyong13579@163.com; 2 Channing Division of Network Medicine, Brigham and Women’s Hospital, Harvard Medical School, Boston, MA 02115, USA; reyhz@channing.harvard.edu; 3 School of Life Sciences, Shanghai University, Shanghai 200444, China; ssdrg@shu.edu.cn; 4 Department of Computer Science, Guangdong AIB Polytechnic College, Guangzhou 510507, China; kyfeng@gdaib.edu.cn; 5 College of Biological and Food Engineering, Jilin Engineering Normal University, Changchun 130052, China; lizd591@jlenu.edu.cn; 6 Bio-Med Big Data Center, CAS Key Laboratory of Computational Biology, Shanghai Institute of Nutrition and Health, University of Chinese Academy of Sciences, Chinese Academy of Sciences, Shanghai 200031, China; 7 CAS Key Laboratory of Tissue Microenvironment and Tumor, Shanghai Institute of Nutrition and Health, University of Chinese Academy of Sciences, Chinese Academy of Sciences, Shanghai 200031, China

**Keywords:** colon immune cell, marker gene, machine learning, feature selection

## Abstract

Immune cell infiltration that occurs at the site of colon tumors influences the course of cancer. Different immune cell compositions in the microenvironment lead to different immune responses and different therapeutic effects. This study analyzed single-cell RNA sequencing data in a normal colon with the aim of screening genetic markers of 25 candidate immune cell types and revealing quantitative differences between them. The dataset contains 25 classes of immune cells, 41,650 cells in total, and each cell is expressed by 22,164 genes at the expression level. They were fed into a machine learning-based stream. The five feature ranking algorithms (last absolute shrinkage and selection operator, light gradient boosting machine, Monte Carlo feature selection, minimum redundancy maximum relevance, and random forest) were first used to analyze the importance of gene features, yielding five feature lists. Then, incremental feature selection and two classification algorithms (decision tree and random forest) were combined to filter the most important genetic markers from each list. For different immune cell subtypes, their marker genes, such as KLRB1 in CD4 T cells, RPL30 in B cell IGA plasma cells, and JCHAIN in IgG producing B cells, were identified. They were confirmed to be differentially expressed in different immune cells and involved in immune processes. In addition, quantitative rules were summarized by using the decision tree algorithm to distinguish candidate immune cell types. These results provide a reference for exploring the cell composition of the colon cancer microenvironment and for clinical immunotherapy.

## 1. Introduction

The large intestine is the last section of the gastrointestinal tract, ending with the anal canal [1]. It is composed of four parts, the cecum, colon, rectum, and anal canal [2], and its main function is to receive food from the small intestine and dehydrate it to form stool. The colon, as the most important section of the large intestine, is responsible for absorbing remaining nutrients and water and delivering feces for excretion [3,4]. As a unique environment with immune tolerance, the colon contains a diverse community of microbes [5] that are associated with various disease states. The microbial community, also known as the microbiome, interacts with the host immune system and has been shown to remodel the immune microenvironment of the human colon [6].

Immune cell profiling may be associated with the physiology and pathology of colon tissue. However, the heterogeneous and dynamic nature of immune cells often hinders the accurate analysis of immune cell profiles using traditional experimental methods. Moreover, the limitations on the number of markers and the low resolution of traditional experimental methods complicate the identification process. With the development of single-cell sequencing, we can now recognize the transcriptomic profiling of all immune cells. Traditionally, the identification of different cell types requires specific biomarkers qualitatively. For instance, CD4 and CD8 are used to differentiate mature T cells [7,8]. However, single-cell sequencing biomarkers cannot (1) quantitatively reveal the differences between different immune cell types or (2) recognize novel biomarkers with great biological significance.

In this study, we utilized single cell RNA sequencing data of a normal gut from the Gut Cell Atlas (https://www.gutcellatlas.org/, assessed on 28 December 2020) [9,10]. We profiled immune cells from 25 candidate immune cell types. Each cell is represented by more than 22,000 genes. We applied several machine learning algorithms to such data for mining important information. The algorithms included five feature ranking algorithms (least absolute shrinkage and selection operator (LASSO) [11], light gradient boosting machine (light GBM) [12], Monte Carlo feature selection (MCFS) [13], minimum redundancy maximum relevance (mRMR) [14], and random forest (RF) [15]), an incremental feature selection (IFS) method [16], a synthetic minority oversampling technique (SMOTE) [17] and two classification algorithms (decision tree (DT) [18] and random forest (RF) [19]). The purpose was to (1) identify potential biomarkers to distinguish different cell types qualitatively and (2) quantify the differences between different cell types. Machine learning models have been summarized to be effective for the prediction and reconstruction of metabolic pathways [20], including a series of reference-based approaches like BlastKOALA [21], KAAS [22], GhostKOALA [21] and RAST [23]. The utilization of machine learning models on metabolic pathway predication and annotation can not only enable low-cost automatic biological function annotation, but also reveal the internal relationships between different biological pathways. Overall, our study reveals cell-type specific biomarkers for immune cell profiling, providing a reference for further monitoring of pathological cell-type composition and exploration of molecular alterations.

## 2. Materials and Methods

**Figure 1:**
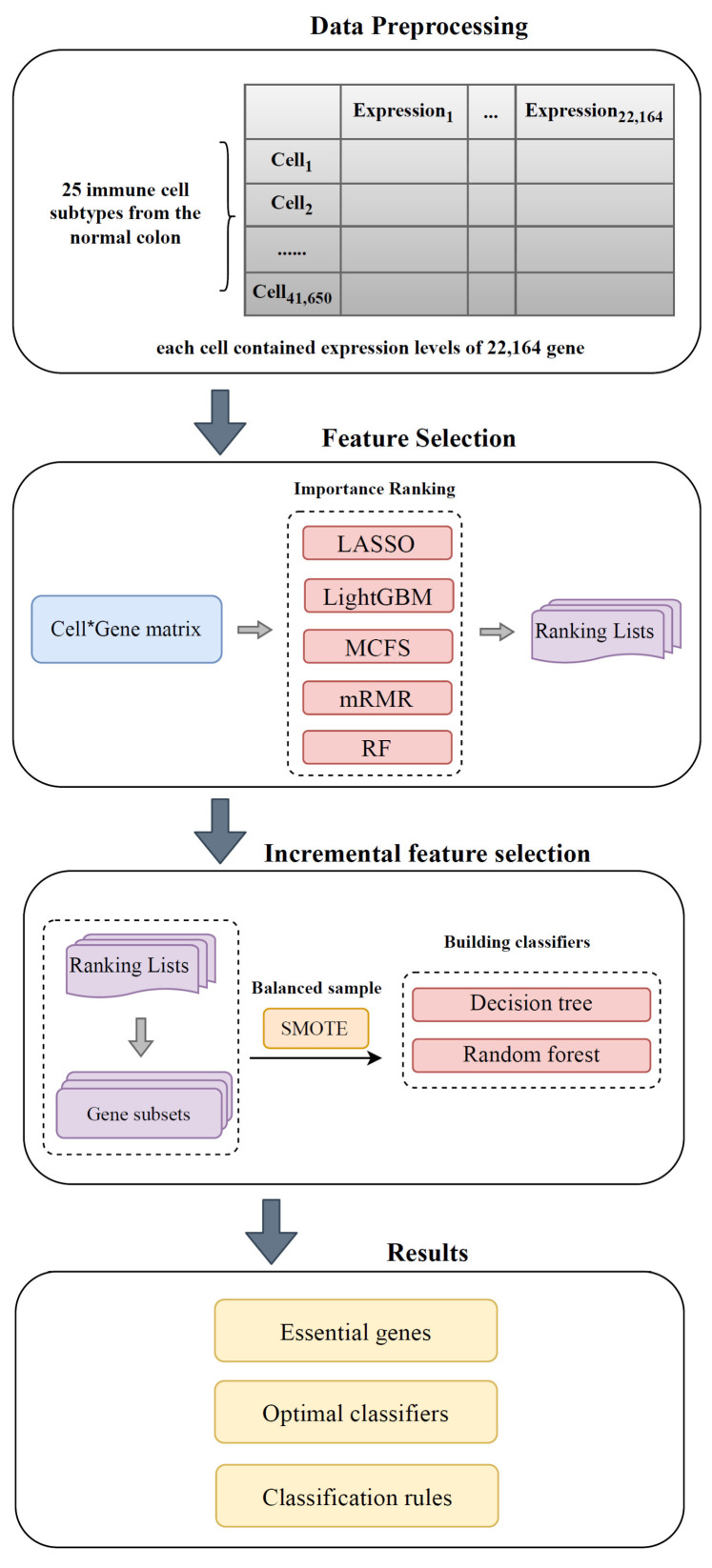
Flow chart of the entire analysis process. scRNA-seq data from 25 immune cells in the normal colon are analyzed by using a machine learning-based approach. The dataset was grouped in accordance with the subtype of immune cells. Gene expression levels were analyzed by five feature selection methods, namely, LASSO, LightGBM, MCFS, mRMR, and RF. The obtained feature lists were fed into the IFS method, which combines DT and RF algorithms, to extract key genes and construct efficient classifiers and classification rules.

Figure 1 demonstrates the machine learning-based pipeline designed in this study. The expression levels of genes from immune cells in normal colon were used as features, which were ranked by five feature-ranking algorithms based on the degree of correlation between genes and cell types. The resulting feature lists were fed into the IFS framework one by one to filter out the most critical subset of genes that can efficiently classify cells into different types. As a result, efficient classifiers and rules were constructed.

### 2.1. Data

The scRNA-seq data of normal colon obtained by James KR et al. [9] were divided into 25 groups based on immune cell subtypes. The dataset comprised a total of 41,650 cells, each with expression levels of 22,164 genes. Table 1 shows the 25 cell subtypes and sample sizes for each immune cell subtype. The sample size of each immune cell type varied greatly, and the dataset needed to be balanced.

### 2.2. Feature Ranking Algorithms

Each cell contained 22,164 genes at the expression level as features. A large number of these genes were not associated with immune cell-specific expression, and feature selection was performed using five feature ranking algorithms. The genes were sorted on the basis of their degree of association with immune cell subtypes. The five ranking methods were LASSO [11], LightGBM [12], MCFS [13], mRMR [14], and RF [15]. These methods have been successfully applied in machine learning applications in the life sciences [24,25,26,27,28,29,30,31].

#### 2.2.1. Last Absolute Shrinkage and Selection Operator

LASSO [11] generates a sparse model that more easily explains the ability of features to contribute to the model. It optimizes a loss function with an L1 regularization term to solve the overfitting problem that tends to arise in linear models. Irrelevant features are removed by penalizing the coefficients of unimportant features to zero, saving computational effort. Features with comparable correlation are assigned similar coefficients, which can alleviate the covariance problem. Features with higher coefficients are considered more important. Thus, features can be ranked in a list in decreasing order of their coefficients. Here, the program of LASSO collected in Scikit-learn (version 1.2.0) [32] was adopted (accessed on 20 January 2023), which was implemented by python (version 3.10). It was performed with default parameters.

#### 2.2.2. Light Gradient Boosting Machine

LightGBM [12] is implemented on the basis of the principles of the gradient-boosted DT method. It introduces a histogram algorithm that binds the dataset into intervals rather than traversing the entire dataset. Gradient-based one-sided sampling and bucketing are used in constructing the tree. It uses a leafwise form when making judgments at the nodes of the tree and only continues the branches with the greatest differentiation. All these features make LightGBM more efficient to train and take up less memory compared with other methods. The more a feature contributes to constructing the trees, the more important it is to the classification task. The features are ranked in a list according to their used times in constructed trees. The current study adopted the LightGBM python program available at https://lightgbm.readthedocs.io/en/latest/ (accessed on 10 May 2020). It was also executed with default parameters.

#### 2.2.3. Monte Carlo Feature Selection

MCFS [13] samples the feature set and the training sample set in a repeated random sampling fashion. For each feature subset, their performance when using different training sample sets is evaluated. The relative importance score of each feature is calculated by combining its contribution to different models. The importance ranking of the features is completed on the basis of this criterion. The MCFS program used in this study was obtained at http://www.ipipan.eu/staff/m.draminski/mcfs.html (accessed on 4 June 2019). It was implemented in Java software dmLab (version 2.1.1). Default parameters were used.

#### 2.2.4. Minimum Redundancy Maximum Relevance

mRMR [14] considers the degree of overlap between features and the degree of correlation between features and target variables. Considering the information overlap of similar features, the algorithm considers that similar features should not be selected simultaneously. mRMR selects the features that correlate most with the target variables and the least redundancy between already selected features. This principle improves the efficiency and accuracy of selecting the best feature set. The mRMR selects features one by one. The selected feature in each round must satisfy the following conditions: (1) maximum correlation to target variable; and (2) minimum redundancies to already-selected features. Based on the selection order, a feature list can be built. Here, the mRMR program available at http://home.penglab.com/proj/mRMR/ (accessed on 2 May 2018) was used, which was implemented by C++. It was also performed using default parameters.

#### 2.2.5. Random Forest

RF uses the reciprocal feature importance measure to rank features. This concept was first introduced by Breiman for RF [19] and later extended to other algorithms by Fisher, Rudin, and Dominici [15]. RF constructs the tree by randomly selecting a subset of features. For each feature that is drawn away, the algorithm compares the performance of the model before and after the extraction, and features that have a greater effect on accuracy are considered more important. The present study used the corresponding package collected in Scikit-learn (version 1.2.0) [32], which was implemented by python (version 3.10). Also, default parameters were adopted.

The above five feature ranking algorithms were applied to the scRNA-seq data mentioned in Section 2.1. Accordingly, five feature lists were obtained, which were called LASSO, LightGBM, MCFS, mRMR, and RF feature lists, respectively.

### 2.3. Incremental Feature Selection

From the five feature lists yielded by five feature ranking algorithms, it was still difficult to extract essential gene features as the threshold of rank was not easy to determine. In view of this, the IFS method was employed [16], which is a widely used method to determine the optimal features for a given classification algorithm. For a feature list, it creates a series of feature subsets with an equal interval *s*. In detail, the first *s* features in the list comprise the first feature subset. Then, the following *s* features are added to the first subset to build the second feature subset. Generally, the following *s* features are added to the current subset to construct the next subset. On each constructed feature subset, a classifier is built with a given classification algorithm. All classifiers are evaluated using a cross-validation method [33]. After assessing the predicted results of cross-validation, the classifier with the best performance can be obtained. Such classifier was called the optimal classifier, whereas features used in this classifier were called optimal features, which comprised the optimal feature subset.

### 2.4. Synthetic Minority Oversampling Technique

As listed in Table 1, the sample sizes of the 25 immune cell types varied dramatically, with B cell (IgA plasma) having 1252 times the sample size of lymphoid DC. This severe imbalance can skew the predictions of the model toward the category with a larger sample size. This study used SMOTE method [17] to tackle this problem by increasing the samples of minority categories. SMOTE maps the training samples into the feature space and generates several new samples for a minority category by concatenating it with one of its nearest samples in the same category. For each minority category, the above procedure was executed several times until it contains as many samples as those in the largest category. This operation is conducted on each category except the largest category, thereby balancing the dataset. The SMOTE python package used in this study was sourced from https://github.com/scikitlearn-contrib/imbalanced-learn (accessed on 24 March 2020), which was performed with its default parameters.

### 2.5. Classification Algorithm

In the IFS method, one classification algorithm is necessary. Its optimal features can be extracted through IFS. Here, two classification algorithms were attempted, DT [18] and RF [19].

#### 2.5.1. Decision Tree

The DT algorithm [18] is a basic, transparent classification algorithm that constructs a tree structure. The algorithm starts at the root node, and a series of recursive operations are performed to reach the leaf node, which contains the category labels. Several attribute judgments are made on the instances between the leaf node and the root node, and the instances are assigned to the downstream subsets based on the judgment results. In this study, the attribute judgments are based on the expression levels of key genes of immune cells.

#### 2.5.2. Random Forest

The RF algorithm [19] judges the class of instances by constructing several DT classifiers and combining the classification results of all DT classifiers in a voting manner. Generally, the RF algorithm has better generalization ability and robustness than the DT algorithm and is more inclusive of data containing noise.

The above DT and RF algorithms were implemented by corresponding packages in Scikit-learn (version 1.2.0) [32] (accessed on 20 January 2023). They were all implemented by python (version 3.10). For convenience, they were performed with default parameters.

### 2.6. Performance Evaluation

In binary classification, precision, recall, and F1-measure [34,35,36,37,38,39,40] are important metrics to evaluate the performance of classifiers. For multi-class classification, these measurements can be defined on each class. The precision, recall, and F1-measure for the *i*-th class can be computed by

(1) $$Precision_{i}=\frac{TP_{i}}{TP_{i}+FP_{i}},$$

(2) $$Recall_{i}=\frac{TP_{i}}{TP_{i}+FN_{i}},$$

(3) $$F1-measure_{i}=\frac{2\times Recall_{i}\times Precision_{i}}{Recall_{i}+Precision_{i}},$$

where $TP_{i}$, $FP_{i}$, and $FN_{i}$ represent true positives, false positives, and false negatives for the *i*-th class. The average of F1-measure on all classes can be used to evaluate the overall performance of classifiers, which is generally called macro F1. However, such measurement may lead to biased results when dealing with imbalanced datasets, where the sample sizes of different classes vary widely. Therefore, a weighted version of F1-measure, termed as weighted F1, is designed, which can be computed by

(4) $$Weighted\;F1=\sum_{i=1}^{L}F1-measure_{i}\times w_{i},$$

where L represents the number of classes (*L* = 25 in this study) and $w_{i}$ represents the proportion of samples in that category to the overall sample. As weighted F1 is more accurate than macro F1, it was selected as the key measurement in this study.

In addition, accuracy (ACC) and Matthew correlation coefficients (MCC) [41] were also employed, which are widely used to measure the performance of classifiers. ACC is defined as the proportion of correctly predicted samples among all samples. The calculation of MCC is more complicated. Two matrices X and Y should be constructed first, which store the true and predicted class of each sample. Then, MCC can be computed by

(5) $$\mathrm{MCC}=\frac{\mathrm{cov}\left(X,Y\right)}{\sqrt{\mathrm{cov}\left(X,X\right)\mathrm{cov}\left(Y,Y\right)}}$$

where cov(X,Y) stands for the correlation coefficient of two matrices.

## 3. Results

The scRNA-seq data from 25 classes of immune cells in normal colon was deeply investigated in this study, where each cell was represented by the expression level of 22,164 genes. Five feature ranking algorithms were used to rank gene features according to their degree of association with subtypes of immune cells, and the algorithms outputted five feature lists (Table S1). As these algorithms are designed following different principles, they can overview the scRNA-seq data from different points of views. Thus, the above five lists were quite different. Generally, the higher ranked gene features have a greater variation in expression levels in different immune cell subtypes from a certain aspect, implying a greater contribution when making judgments about unknown cells.

### 3.1. Dynamics of Classifier Performance

**Figure 2:**
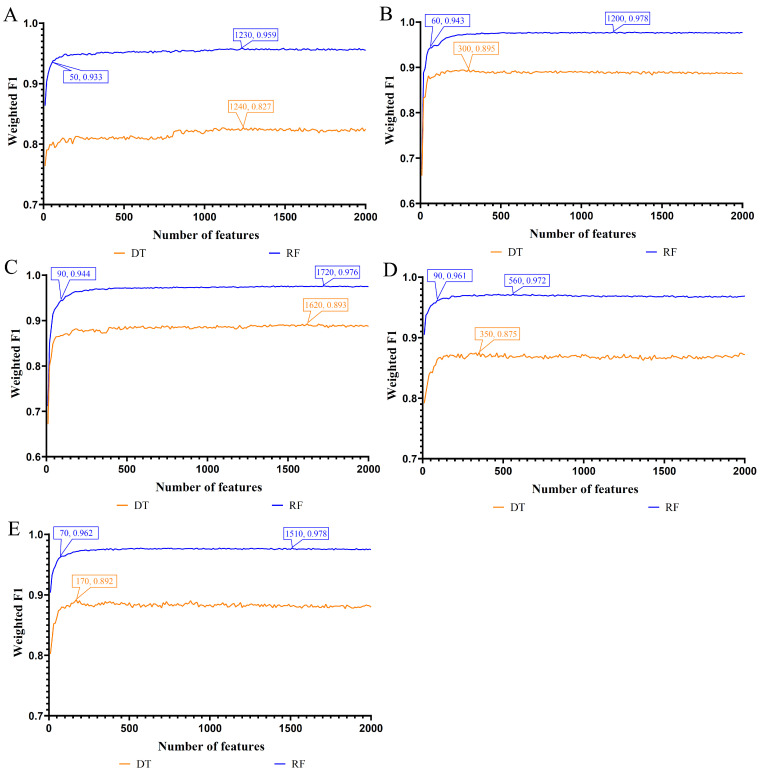
IFS curves for evaluating the performance of the two classification algorithms based on weighted F1. (**A**). IFS curves based on the LASSO feature list. (**B**). IFS curves based on the LightGBM feature list. (**C**). IFS curves based on the MCFS feature list. (**D**). IFS curves based on the mRMR feature list. (**E**). IFS curves based on the RF feature list. On each curve, the best performance is marked, while the relative high performance based on a few features is also marked on the curve of RF.

As listed in Table S1, each list contained lots of gene features. If all features were considered, the running time of IFS methods would be very long. On the other hand, the essential genes that are highly related to the classification of immune cells generally occupy a small proportion of all genes. Thus, to save time, only the top 2000 gene features in each list were considered, which were fed into the IFS method. Setting the interval to 10, 200 gene subsets were constructed from each list. For each gene subset, DT and RF were adopted to construct classifiers, and each classifier was evaluated by 10-fold cross-validation. The cross-validation results were counted as macro F1, weighted F1, ACC, and MCC, which are available in Table S2. Furthermore, the ACC for each fold is also provided in Table S2. To display the performance change in classifiers with different feature subsets, some IFS curves were plotted, as shown in Figure 2, where weighted F1 was defined as *Y*-axis and the size of the subset was set as the *X*-axis.

When DT was used in the IFS method, its highest weighted F1 values under the five feature lists were 0.827, 0.895, 0.893, 0.875, and 0.892, respectively. Such performance was obtained using the top 1240, 300, 1620, 350, and 170 features in five feature lists, respectively. Accordingly, the optimal DT classifiers can be constructed on each feature list using the above top features in the corresponding feature lists. Their detailed performance is listed in Table 2. These classifiers all have good performance for classification of immune cells.

Additionally, RF was also attempted in the IFS method. According to Figure 2, the highest weighted F1 values under the five feature lists were 0.959, 0.978, 0.976, 0.972, and 0.978, respectively. The top 1230, 1200, 1720, 560, and 1510, respectively, gene features in five feature lists were used to access such performance. Evidently, such performance was higher than that of DT. Likewise, with above features, five optimal RF classifiers can be set up. The detailed performance of these five classifiers is also listed in Table 2. Clearly, their performance was extreme high and all measurements were higher than 0.9.

Based on the above results, we can find that the optimal RF classifiers on the LightGBM and RF feature lists were better than other optimal RF/DT classifiers. These two classifiers can be efficient tools for classifying immune cells.

### 3.2. Relationships between the Most Essential Genes Extracted from Five Lists

**Figure 3:**
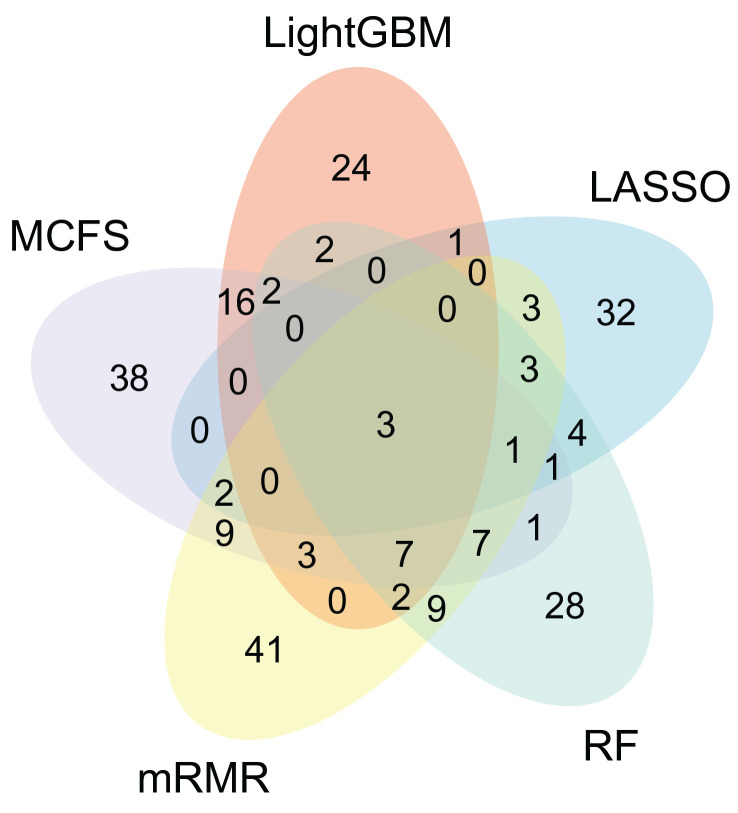
Venn diagram of the most essential features extracted from the lists yielded by LASSO, LightGBM, MCFS, mRMR, and RF. The overlapping circles indicate genes that are identified as the most essential genes by multiple ranking algorithms.

From the results in Section 3.1, the optimal RF classifier was much better than the optimal DT classifiers on the same feature list. Thus, we selected the optimal features for RF in five lists for further analysis. However, the number of optimal features for RF were always too many to give a detailed investigation. For example, there were 1230 optimal features for the optimal RF classifier on the LASSO feature list. In view of this, we must extract the most essential genes from each optimal feature subset. By checking the IFS results (Table S2), it can be found that some RF classifiers provided a little lower performance than the optimal RF classifiers, but they needed much less gene features. We refer to these classifiers as feasible classifiers. The weighted F1 of these feasible classifiers are marked on the IFS curves, as shown in Figure 2, and their detailed performance is listed in Table 2. The feasible RF classifiers on five feature lists used the top 50, 60, 90, 90, and 70 features in the corresponding lists. The relationship between the above five gene subsets is illustrated by a Venn diagram, as shown in Figure 3. The detailed intersection results can be found in Table S3. It can be observed that some genes were identified as the most essential genes by multiple feature ranking algorithms, implying they were more likely to be the biomarker genes. More attention should be paid to these genes. As for those identified by one feature ranking algorithm, we cannot deny their possibility to be latent biomarker genes. Thus, we listed all of them in Table S3, which may give useful insights for other investigators.

**Figure 4:**
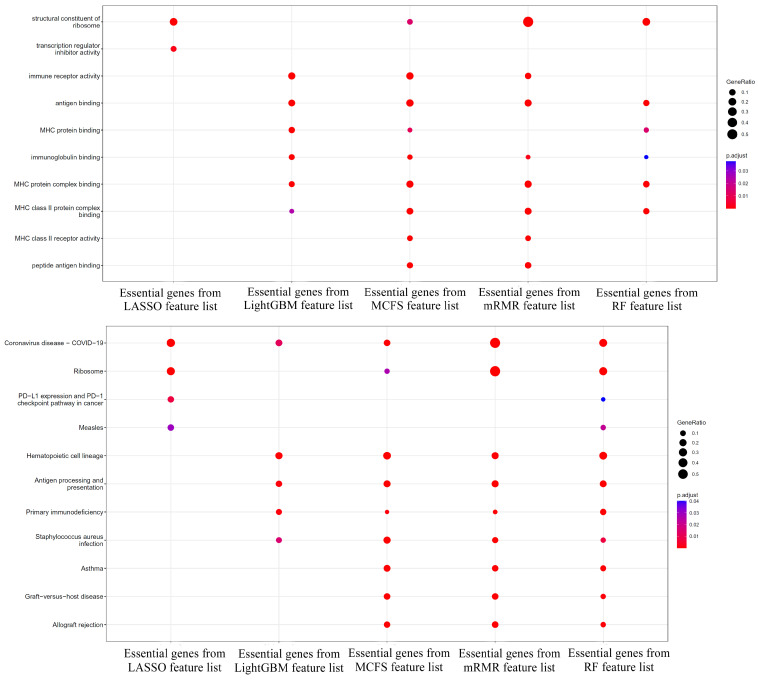
Enrichment results on essential genes extracted from five lists. The above figure shows the enriched gene ontology terms and the below figure shows the enriched KEGG pathways.

Furthermore, the enrichment analysis was conducted on above five gene subsets. The enriched gene ontology (GO) terms and KEGG pathways for each subset are illustrated in Figure 4. The GO enrichment results showed that the genes of different subsets always performed the same molecular function. One of the most popular GO terms including GO:0003735, GO:0003823, and GO:0023023 were enriched by at least four gene subsets. GO:0003735 describes the structural constituent of ribosomes. In colorectal cancer, increased protein synthesis may be a marker of rapid proliferation and growth of cancer cells. In colorectal cancer, increased protein synthesis may be a marker for rapid proliferation and growth of cancer cells, and abnormalities in ribosome biosynthesis and function may be associated with tumorigenesis [42,43]. GO:0003823 describes antigen binding. Antigen binding is directly related to the immune response. Colon and colorectal cancer cells may express different tumor antigens that can be recognized by the immune system. Therefore, antigen binding may be associated with tumor immune escape or immunotherapy [44]. GO:0023023 describes MHC protein complex binding. The major histocompatibility complex (MHC) plays a key role in the immune response, especially in the cell-mediated immune response. Aberrant expression or modification of MHC proteins may be associated with immune escape of tumor cells, especially in colorectal cancer [45]. MHC proteins are also involved in regulating the function of tumor-infiltrating lymphocytes during antigen processing [46]. KEGG results likewise showed that different sets of genes shared multiple pathways. One of the popular pathways contained hsa03010, hsa04612, etc. hsa03010 describes ribosome and hsa04612 describes antigen processing and presentation. KEGG results are highly similar to those of GO enrichment results, emphasizing the role of ribosome and antigen processing and presentation in the colon immune response.

### 3.3. Classification Rules

**Figure 5:**
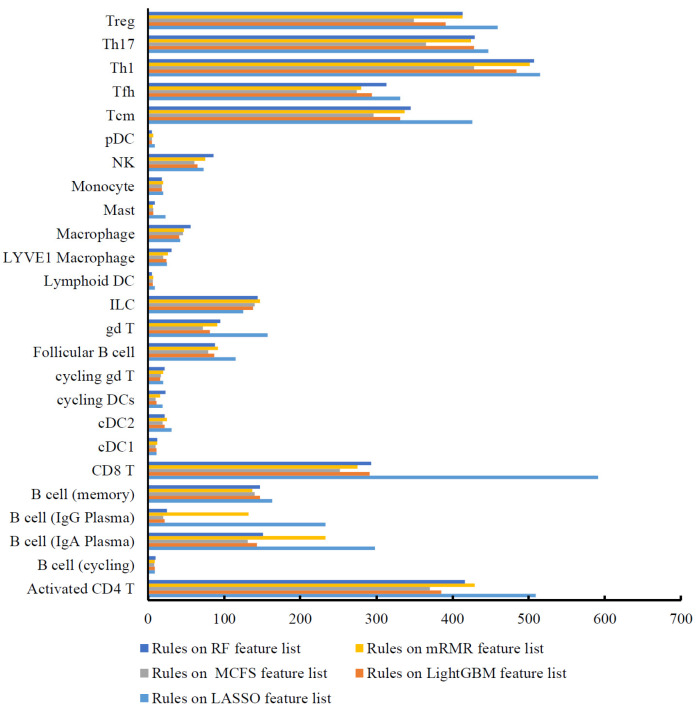
A bar chart to show the number of rules for each immune cell type in five rule groups on five feature lists.

**Figure 6:**
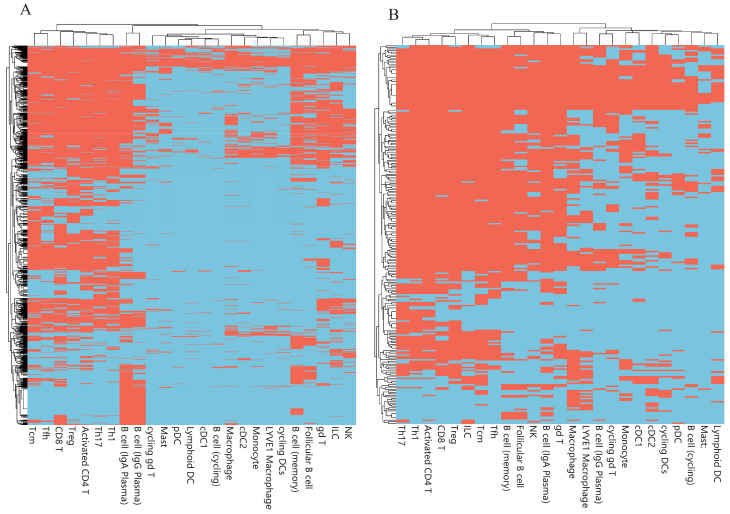
Heatmap for rule-associated genes identified on two feature lists (red for genes selected to predict the cell type in the rule and sky blue for other genes). (**A**). Heatmap based on rules on the LASSO feature list. (**B**). Heatmap based on rules on the LightGBM feature list.

**Figure 7:**
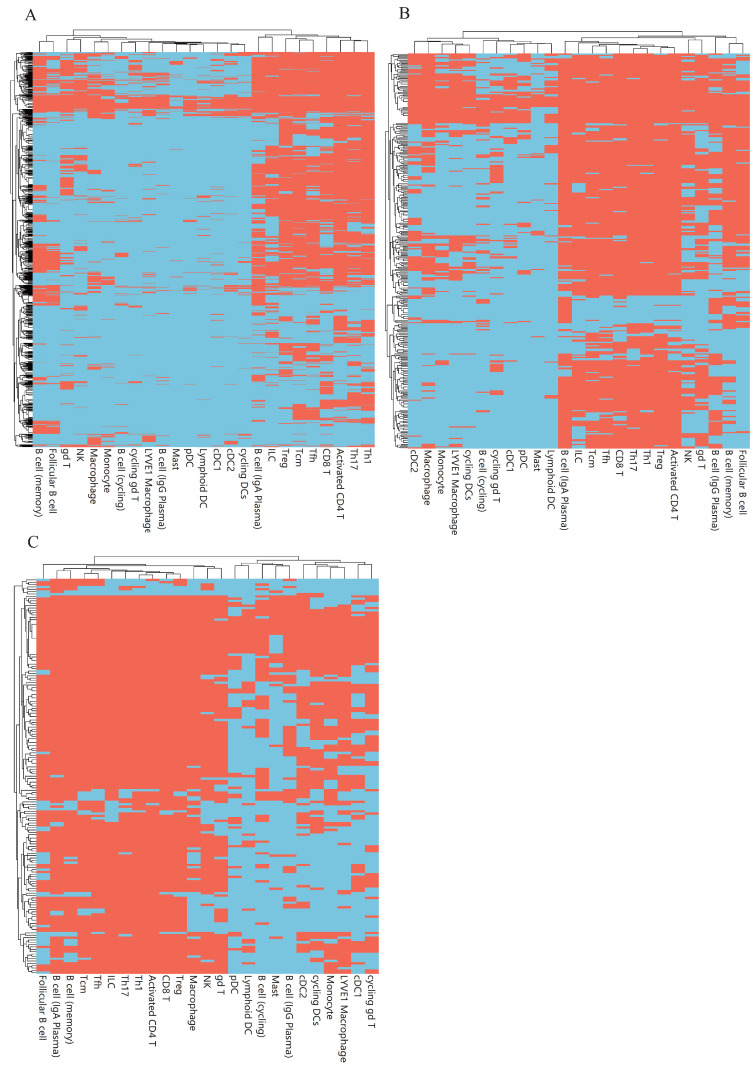
Heatmap for rule-associated genes identified on three feature lists (red for genes selected to predict the cell type in the rule and sky blue for other genes). (**A**). Heatmap based on rules on the MCFS feature list. (**B**). Heatmap based on rules on the mRMR feature list. (**C**). Heatmap based on rules on the RF feature list.

Based on the IFS results, the performance of DT was generally lower than that of RF. However, DT has a special merit that is not shared by RF. From the constructed DT, a group of classification rules can be obtained, each of which indicated a path from the root to one leaf. As mentioned above, the optimal DT classifiers used the top 1240, 300, 1620, 350, and 170 features in the five lists. Based on these features, we constructed five DTs using all cell samples, thereby accessing five rule groups. Table S4 shows these five rule groups, which contained 4660, 3457, 3143, 3761, and 3665 rules. Any rule group can be used to classify immune cells. Furthermore, as each rule contained a number of genes and thresholds on the expression level of involved genes, it can indicate the expression pattern of genes for one cell type. In each rule group, any cell type received one or more rules. The number of rules for each cell type in five groups are illustrated in Figure 5. It can be observed that some cell types (e.g., Treg, Th17, Th1, Activated CD4 T, etc.) were assigned more rules than other cell types. For the comparison of classification rules generated on different feature lists for different cell types, we summarized the gene markers present in these rules, termed as rule-associated genes (Figure 6 and Figure 7). Cell types were further clustered on the basis of the presence or absence of these gene markers. The clustering results revealed the molecular similarity between different cell types. As each rule group was generated on the list yielded by different feature ranking algorithms, which offered distinct perspectives, the identified cell-type clusters were different.

## 4. Discussion

Predicting and annotating metabolic pathways has been a time-consuming and expensive task for biological studies. The utilization of machine learning models to reconstruct metabolic pathways provides a novel way to understand the molecular mechanisms for biological processes and reveals internal correlations between different biological functions. Compared to traditional experimental or computational methods, we summarized molecular signatures for specific cell types or the cell-type specific biological functions, providing a higher and quantitative level approach for cell clustering and functional exploration. In this study, we applied several machine learning algorithms to (1) identify biomarkers for distinguishing 25 cell types and (2) establish quantitative classification rules for cell-type prediction. Recent publications have supported the predicted biomarkers and rules, validating the efficacy and accuracy of using machine learning algorithms for cell-type feature recognition. Our analytic results for the 25 cell types were summarized into three major categories: T cell family identification, B cell family identification, and other cells, including innate lymphoid cells. The discussed genes are listed in Table 3.

### 4.1. T Cell Family

The first identified predicted gene is ***KLRB1***, identified by the LASSO. The receptor of *KLRB1* has been reported to interact with and be correlated with CD69 [47], a well-known immune cell biomarker for cultured peripheral T cells [48]. During the activation of T cells, the expression of CD69 is upregulated [49,50]. Therefore, the interactive and correlated receptor *KLRB* can help us distinguish activated CD4 T cells from other cells, validating our prediction.

The next feature ranking algorithm, LightGBM, identified specific genes, such as ***CST3*** and ***AIF1***, which have been predicted to distinguish cell types from the colon transcriptome. *CST3* has been widely observed in mast cells [51]. Therefore, such a gene may help recognize mast cells from other cell types. *AIF1* is a regulatory allograft inflammation initiator [52,53]. As for its cell specificity, *CST3* has been shown to be specifically expressed T cells [54] and fibroblasts [55] to degrade extracellular matrix, remodel immune responses, and regulate tissue development [56]. *AIF1* has been shown to regulate trophoblast differentiation and participate in FasL/CD95L-mediated cell death [57]. Considering that FasL/CD95L is mediated by T cells, the identification of *AIF1* may help recognize T cells, including cytotoxic T cell and T helper cells.

The next algorithm, MCFS, recognized ***JCHAIN*** as a cell-type specific biomarker for prediction, validating the efficacy and accuracy of the newly presented methods. ***HLA-DRA***, another predicted biomarker, has been shown to be specifically expressed in IFN-γ-stimulated cells [58], including CD8 T cell and NK T cells. Therefore, such a gene is definitely a biomarker for cell-type recognition.

The mRMR identified ***TIGIT***, which has been widely recognized in various T cell subtypes [59,60]. Differentially expressed *TIGIT* has been observed in central memory T cells, even compared to other cell types [59,61]. Therefore, regarding *TIGIT* as a potential cell-specific biomarker to distinguish different cell types in colon tissues is reasonable.

For RF, in the summarized quantitative rules, ***RPS12*** has been predicted to be highly expressed in T follicular helper cells. In accordance with recent publications, a dynamic atlas of immune cells recognizes a high expression of *RPS12* in T follicular helper cells [62]. ***MS4A1*** is predicted to be a quantitative biomarker for Th1 cells in our newly established rules, which has also been validated by recent publications [63].

### 4.2. B Cell Family

In the first rule generated on the LASSO feature list, a low expression level of *RPL30* helps us recognize B cell IGA plasma cells. In accordance with recent publications, *RPL13* participates in the reprogramming of immune cells, including B cells, during immune responses [64,65]. A lower expression level of ***RPL30*** has been observed in plasma cells compared with immature B cells, validating our quantitative rule [64,66]. Similarly, a lower expression level of ***ANXA1*** has been confirmed to help recognize B cell IGA plasma cells and B memory cells with low expression levels. *ANXA1* is shown to be highly expressed during cell proliferation [67]. Therefore, using *ANXA1* to eliminate highly proliferative cells from candidates is reasonable.

Among various quantitative rules generated on the LightGBM feature list, ***JCHAIN*** has been shown to be negatively correlated with the production of antibodies, including IgG [68,69,70]. Therefore, establishing the rule that low expression of *JCHAIN* help to recognize IgG-producing B cell is reasonable.

For the results using the MCFS algorithm, we also recognized a low expression of ***JCHAIN*** in B cells (IgA, IgG, and memory B cells) [68,69,70]. Another gene, ***ICA1***, as a hub gene, has been shown to be stably expressed in T regulatory cells and T helper cells [71,72]. In addition, such a gene has been shown to be associated with exhausted CD4 T cell activation during the progression of colorectal cancer [73]. Therefore, recognizing such a gene as a biomarker for T helper cells is reasonable. Another gene, ***TYROBP***, is also a widely reported immune-associated gene. In accordance with recent publications, upregulation of *TYROBP* has been shown to be downregulated in a specific cell type, Lyve1 M2-like macrophage [74], corresponding with the quantitative rule generated on the MCFS feature list.

***RPS27***, as a specifical gene identified by mRMR, has been widely reported to be a housekeeping gene in the colon [75]. However, further studies showed that during pathological immune activation, *RPS27* has shown to be abnormally regulated [75]. Therefore, such a gene may help recognize activated immune cells from our candidates, including plasma B cells and activated CD4 T cells. We also recognized some genes that we have discussed above, including ***JCHAIN*** and ***HLA-DRA***, as candidate biomarkers for cell-type classification.

As for quantitative rules, we also identified genes, such as ***JCHAIN*** and ***HLA-DRA***, as quantitative rule biomarkers. ***IFITM1***, as an interferon-induced transmembrane protein, has been widely reported to be associated with follicular B cells [76,77,78]. In accordance with the quantitative rule we established, a low expression of such a gene was shown in mature follicular B cells during virus infection [79], consistent with recent publications.

The component of immune cells in the colon has been regulated by ***IGHA1*** during ulcerative colitis [80]. B cells uniquely express *IGHA1* during the immune activation procedures. Therefore, recognizing such a gene as a biomarker to distinguish other different cells from B cells is reasonable. Similarly, ***CD37*** regulates T-cell dependent B-cell activation [81]. Therefore, we can distinguish T cells and B cells from other cell types using *CD37*, validating the efficacy and accuracy of the RF feature ranking algorithm in cell-type prediction.

### 4.3. Other Cells

***RPS13*** and ***RPL34*** are both ribosome-associated genes. Ribosome-associated genes are general genes without cell specificity or tissue specificity [82]. However, ribosome-associated proteins have been reported to be activated by natural humoral immune regulation under the pathogenic condition of colon tissue [83]. Therefore, a high expression of ribosome-associated genes may indicate the activation of natural humoral immune responses, involving the recognition of specific cell types, including macrophage, monocytes, and mast cells. Genes, such as ***MALAT1*** and ***TMSB4X***, have been reported to be cell-type specific genes. *MALAT1* is reported to be expressed in dendritic cells [84], helping us identify lymphoid dendritic cells in the colon. *TMSB4X* has been reported to be expressed in monocytes and dendritic cells. Such a gene can also help us distinguish different cell types although with less specificity.

***SOCS3*** as a specific monocyte and macrophage-associated gene is shown to participate in modulating the immune microenvironment of the colon [85,86]. A low expression of SOCS3 reflects the physiological status of monocytes and macrophages [86]. The expression level of such a gene is highly upregulated after immune activation. In addition, ***IL7R*** has been predicted to help recognize gdT cells. In accordance with recent publications, the inhibition of IL-17A protects against thyroid immune-related adverse events mediated by gdT [87]. Therefore, such a gene is a quantitative biomarker to recognize gdT.

***CD-74*** has been recognized in the colon as a potential biomarker for colon cancer [88,89]. Although most function-associated publications on such a gene focused on epithelial cells, such a gene has also been shown to participate in antigen presentation and inflammation regulation [90]. Therefore, using such a gene as a biomarker for dendritic cells (antigen presentation), macrophages (antigen presentation), and T cells (inflammation regulation) is reasonable [89,90].

***RPL28*** has been shown to regulate inflammatory and tumorigenic networks in colon tissue [91,92,93]. *RPL28* is related to the prognosis of colorectal cancer by participating in immune remodulation [92,94]. Innate lymphoid cells can be quantitatively recognized by ***ANKRD28*** and ***TYROBP*** with high expression patterns based on predicted rules. Expression patterns of two such genes in innate lymphoid cells have been validated by recent publications [95,96,97].

As discussed above, we identified a series of biomarkers and several quantitative rules for cell-type classification. Additional features identified by the RF feature ranking algorithm have been validated by other algorithms. Recent publications have supported the features and rules identified by RF, suggesting that it may be the most effective approach for predicting cell types and identifying potential cell biomarkers. Therefore, the machine learning models employed in this study conducted a comprehensive analysis on single-cell level cell classification and provided a novel approach for identifying cell-type biomarkers at the single-cell level.

### 4.4. Limitation of This Study

In this study, several feature ranking algorithms were employed to analyze the scRNA-seq data. It is not certain whether these algorithms can mine all essential genes. Some biomarker genes may still not be discovered, which can be exclusively identified by a certain algorithm. In the future, we will continue this study to design a more perfect method.

## 5. Conclusions

Based on single-cell RNA sequencing data of 25 different types of immune cells from the normal intestine, a machine learning-based pipeline was designed. It identified genetic markers that can qualitatively and quantitatively distinguish between these cell subtypes. Our analysis revealed key genes representing different immune cell subtypes, and we constructed some multi-class classifiers to classify these cell types. We further generated rules for quantifying gene expression differences between immune cell subtypes using the DT algorithm. These results were validated by previously published studies, and they may serve as a useful reference for clinical diagnosis and targeted therapy of colon cancer. The codes used in this study can be accessed at https://github.com/chenlei1982/ColonImmune/.

## Figures and Tables

**Table 1:** Twenty-five immune cell subtypes and sample sizes.

Index	Immune Cell Subtypes	Sample Size
1	Activated CD4 T	1531
2	B cell (cycling)	15
3	B cell (IgA Plasma)	12,522
4	B cell (IgG Plasma)	342
5	B cell (memory)	4508
6	CD8 T	3145
7	cDC1	38
8	cDC2	107
9	cycling DCs	47
10	cycling gd T	25
11	Follicular B cell	2582
12	gd T	548
13	ILC	832
14	Lymphoid DC	10
15	LYVE1 Macrophage	91
16	Macrophage	268
17	Mast	1151
18	Monocyte	98
19	NK	452
20	pDC	13
21	Tcm	3042
22	Tfh	1786
23	Th1	2833
24	Th17	3432
25	Treg	2232

**Table 2:** Performance of some key classifiers under different feature lists and classification algorithms.

Feature List	Classification Algorithm	Number of Features	ACC	MCC	Macro F1	Weighted F1
LASSO feature list	Decision tree	1240	0.823	0.796	0.775	0.827
	Random forest	1230	0.960	0.953	0.953	0.959
	Random forest	50	0.933	0.923	0.937	0.933
LightGBM feature list	Decision tree	300	0.895	0.878	0.871	0.895
	Random forest	1200	0.978	0.974	0.985	0.978
	Random forest	60	0.943	0.934	0.949	0.943
MCFS feature list	Decision tree	1620	0.892	0.875	0.881	0.893
	Random forest	1720	0.976	0.972	0.983	0.976
	Random forest	90	0.944	0.935	0.954	0.944
mRMR feature list	Decision tree	350	0.873	0.853	0.855	0.875
	Random forest	560	0.972	0.968	0.973	0.972
	Random forest	90	0.961	0.955	0.969	0.961
RF feature list	Decision tree	170	0.891	0.874	0.863	0.892
	Random forest	1510	0.977	0.974	0.984	0.978
	Random forest	70	0.962	0.956	0.970	0.962

**Table 3:** Latent biomarker genes identified in this study.

Category	Gene Symbol	Description
T cell family	KLRB1	Killer Cell Lectin Like Receptor B1
	CST3	Cystatin C
	AIF1	Allograft Inflammatory Factor 1
	JCHAIN	Joining Chain of Multimeric IgA and IgM
	HLA-DRA	Major Histocompatibility Complex, Class II, DR Alpha
	TIGIT	T Cell Immunoreceptor with Ig and ITIM Domains
	RPS12	Ribosomal Protein S12
	MS4A1	Membrane Spanning 4-Domains A1
B cell family	RPL30	Ribosomal Protein L30
	ANXA1	Annexin A1
	JCHAIN	Joining Chain of Multimeric IgA and IgM
	ICA1	Islet Cell Autoantigen 1
	TYROBP	Transmembrane Immune Signaling Adaptor TYROBP
	RPS27	Ribosomal Protein S27
	HLA-DRA	Major Histocompatibility Complex, Class II, DR Alpha
	IFITM1	Interferon Induced Transmembrane Protein 1
	IGHA1	Immunoglobulin Heavy Constant Alpha 1
	CD37	CD37 Molecule
Other cells	RPS13	Ribosomal Protein S13
	RPL34	Ribosomal Protein L34
	MALAT1	Metastasis Associated Lung Adenocarcinoma Transcript 1
	TMSB4X	Thymosin Beta 4 X-Linked
	SOCS3	Suppressor Of Cytokine Signaling 3
	IL7R	Interleukin 7 Receptor
	CD-74	CD74 Molecule
	RPL28	Ribosomal Protein L28
	ANKRD28	Ankyrin Repeat Domain 28
	TYROBP	Transmembrane Immune Signaling Adaptor TYROBP

## Data Availability

The data presented in this study are openly available at https://www.nature.com/articles/s41590-020-0602-z (assessed on 28 December 2020), reference number [9].

## Supplementary Materials

The following supporting information can be downloaded at: https://www.mdpi.com/article/10.3390/life13091876/s1, Table S1: Feature ranking results obtained by LASSO, LightGBM, MCFS mRMR, and RF. Table S2: IFS results with different classification algorithms. Table S3: Intersection of the most essential genes extracted from five lists yielded by LASSO, LightGBM, MCFS, mRMR, and RF. The features that appear in the five, four, three, two, and one feature subsets are shown. Table S4. Classification rules generated by the DT classifier on its optimal features in each list.
